# Impact of COVID-19 Vaccination on Hospitalization and Mortality: A Comparative Analysis of Clinical Outcomes During the Early Phase of the Pandemic

**DOI:** 10.3390/idr17040074

**Published:** 2025-06-27

**Authors:** Brenda Garduño-Orbe, Paola Selene Palma-Ramírez, Eduardo López-Ortiz, Gabriela García-Morales, Juan Manuel Sánchez-Rebolledo, Alexis Emigdio-Loeza, Anel Gómez-García, Geovani López-Ortiz

**Affiliations:** 1 Unidad de Medicina Familiar Número 26, Instituto Mexicano del Seguro Social, Acapulco 39700, Guerrero, Mexico; brenda.gardunoo@imss.gob.mx (B.G.-O.); gabriela.garciamora@imss.gob.mx (G.G.-M.); juan.sanchezre@imss.gob.mx (J.M.S.-R.); 2 Unidad de Medicina Familiar No. 29 con UMAA, Instituto Mexicano del Seguro Social, Acapulco 39906, Guerrero, Mexico; 10064039@uagro.mx; 3 Subdivisión de Medicina Familiar, Facultad de Medicina, Universidad Nacional Autónoma de México, Ciudad Universitaria 04510, Ciudad de México, Mexico; eduardolopezepi@comunidad.unam.mx; 4 Unidad Médica de Alta Especialidad Número 1, Hospital de Especialidades Centro Médico Nacional del Bajío, Instituto Mexicano del Seguro Social, León de los Aldama 37320, Guanajuato, Mexico; a.emigdioloeza@ugto.mx; 5 Centro de Investigación Biomédica de Michoacán, Instituto Mexicano del Seguro Social, Morelia 58351, Michoacán, Mexico; anel.gomez@imss.gob.mx

**Keywords:** COVID-19, SARS-CoV-2, vaccination, hospitalization, mortality, comorbidity, Mexico

## Abstract

**Background**: Although COVID-19 vaccination has been effective in reducing severe illness and mortality, its differential clinical behavior in vaccinated and unvaccinated individuals during the early stages of the pandemic—especially in settings with partial coverage and real-world conditions—remains insufficiently characterized. **Objective**: To assess differences in clinical presentation, comorbidity prevalence, hospitalization, and mortality between vaccinated and unvaccinated patients diagnosed with SARS-CoV-2 during the early phase of the pandemic. **Methods**: An analytical cross-sectional study was conducted using 4625 electronic medical records of patients diagnosed with COVID-19 in Guerrero, Mexico, between 1 January and 31 December 2021. Variables included vaccination status, age, sex, comorbidities, symptom severity, clinical outcomes, and mortality. Statistical analyses involved chi-square tests, logistic regression for hospitalization probability, and Cox proportional hazards models for mortality risk. **Results**: Of the patients analyzed, 31.45% had received at least one vaccine dose. Fever, headache, cough, and anosmia were more frequent among vaccinated individuals (*p* < 0.001). Prostration and chest pain were strongly associated with hospitalization in both groups. In unvaccinated patients, smoking (OR = 4.75), obesity (OR = 3.85), and hypertension (OR = 2.94) increased hospitalization risk. Among vaccinated patients, diabetes mellitus (OR = 3.62) and hypertension (OR = 2.88) were key predictors. Vaccination was significantly associated with lower odds of hospitalization (OR = 0.38; 95% CI: 0.26–0.55) and reduced mortality risk (HR = 0.24; 95% CI: 0.08–0.71). **Conclusions**: Vaccination status was a significant protective factor for both hospitalization and mortality; however, clinical symptoms and comorbidity-related risks varied, highlighting the need for individualized patient management strategies.

## 1. Introduction

The SARS-CoV-2 pandemic had significant effects across all levels of society, particularly on healthcare systems, prompting the accelerated development of vaccines to reduce COVID-19-related morbidity and mortality [1,2]. Although vaccination has marked a significant advancement in disease prevention, its effectiveness at the population level has shown substantial heterogeneity, often linked to structural inequalities, including limited access to healthcare services [3,4,5,6].

The efficacy of COVID-19 vaccines in preventing severe illness and death has been extensively documented in clinical trials and observational studies [7,8]. However, heterogeneity in the availability and distribution of different vaccines across countries and regions led to complex epidemiological scenarios requiring specific analyses [5,6]. This variability has resulted in clinical differences between populations, highlighting the need for detailed monitoring of epidemics at the local level [9,10].

COVID-19 vaccines elicit a robust immune response against SARS-CoV-2, which may alter the clinical presentation of the disease [11,12]. Several studies have reported differences in symptoms between vaccinated and unvaccinated patients; however, findings have been inconsistent, partly due to variability in immune profiles related to the spatiotemporal dynamics of SARS-CoV-2 variants [13,14,15]. Likewise, the impact of preexisting comorbidities on the clinical course of COVID-19 may be significantly modified in the presence of vaccine-induced immunity, a key aspect for risk stratification and individualized clinical management [16,17].

Moreover, specific groups, such as older adults and individuals with chronic diseases, may exhibit suboptimal immune responses to vaccination [18,19]. This underscores the need to further characterize the clinical presentation and disease progression within these subgroups, particularly in settings where public health strategies, limited vaccine access, and socioeconomic conditions shaped the epidemiological landscape during the early phases of the pandemic.

In Mexico, the response to the health emergency faced multiple structural and operational challenges, including limited hospital capacity, supply shortages, and fragmented national coordination [20]. Genomic surveillance data indicate that the B.1.1.519 variant was predominant in Mexico during the first half of 2021, followed by the Delta variant (B.1.617.2) in the second half [21]. Although the initial availability of vaccines marked a substantial advancement, implementation was constrained by logistical barriers, regional disparities in distribution, and deep-rooted inequities in healthcare access [22,23,24]. These factors may have influenced the clinical course of the disease, particularly among vulnerable groups. Therefore, analyzing differences based on vaccination status is essential to understand better the clinical behavior of COVID-19 at the regional level.

Based on these considerations, this study aimed to characterize differences in clinical presentation, prevalence of comorbidities, hospitalization rates, and mortality between vaccinated and unvaccinated patients diagnosed with SARS-CoV-2 during the early phase of the pandemic.

## 2. Materials and Methods

An analytical cross-sectional study was conducted. Electronic medical records from both primary and secondary care levels were reviewed for patients affiliated with a family medicine unit in Acapulco, Guerrero, Mexico. All records with a confirmed diagnosis of COVID-19, validated by a positive RT-PCR or rapid antigen test, between 1 January and 31 December 2021, were included. Diagnostic confirmation of SARS-CoV-2 infection was based on institutional records indicating a positive result from either a rapid antigen test or reverse transcription polymerase chain reaction (RT-PCR). RT-PCR was performed on nasopharyngeal swab specimens using standardized molecular protocols authorized by the national health authority. The procedure involved RNA extraction, amplification of viral target genes (such as N, E, or RdRp), and detection via real-time fluorescence [25]. Rapid antigen tests were lateral flow immunoassays designed to detect the viral nucleocapsid protein and were administered by trained personnel following the manufacturer’s instructions and institutional guidelines [26].

This analysis comprised the full census of confirmed COVID-19 cases managed at the selected medical unit during the study period. Incomplete records or those containing reporting errors were excluded from the analysis.

The variables assessed included age, sex, history of COVID-19 vaccination (number of doses, administration dates, and vaccine brand), clinical history, comorbidities (hypertension; diabetes; asthma; COPD; immunosuppression; smoking; HIV/AIDS; cancer; obesity; chronic cardiac, renal, and hepatic diseases; hemolytic anemia; neurological disorders; and tuberculosis), clinical manifestations (respiratory and systemic symptoms), treatment received (including supplemental oxygen administration and use of antivirals), and clinical outcomes such as hospitalization, admission to the intensive care unit, and time elapsed between symptom onset, medical care, and, where applicable, death.

### Statistical Analysis

The Kolmogorov–Smirnov test was used to assess the normality of data distribution. Age did not follow a normal distribution; therefore, the Mann–Whitney U test and Welch’s *t*-test were applied due to unequal variances between groups. Data are presented as median (minimum–maximum) and, when appropriate, as mean ± standard deviation.

The chi-square (χ^2^) test and Fisher’s exact test were used to analyze differences between categorical variables (vaccinated vs. unvaccinated), symptoms, and comorbidities. Additionally, a logistic regression model was fitted with hospitalization as the dependent variable, adjusted for age, sex, and vaccination status.

To assess the probability of hospitalization based on symptoms and comorbidities, a logistic regression analysis, stratified by vaccination status, was performed to investigate. The Nagelkerke R^2^ was reported to assess the model’s goodness of fit and predictive power. A Cox proportional hazards model, adjusted for age (continuous), was conducted to evaluate the association between vaccination status and time to death among confirmed COVID-19 cases, using the subset of deceased individuals with valid dates. Statistical analyses were conducted using SPSS (Version 23.0, IBM Corp., Armonk, NY, USA) and GraphPad Prism (Version 5.0, GraphPad Software, San Diego, CA, USA). Additionally, the scipy.stats, matplotlib, and pandas libraries in the Python 3.9 environment were used for data visualization and complementary analyses.

The study was approved by the Local Research and Research Ethics Committee 1101 of the Decentralized Administrative Operation Body of the Mexican Social Security Institute (IMSS) in the state of Guerrero. The institutional registry number is R-2022-1101-001.

## 3. Results

A total of 4625 medical records of individuals diagnosed with SARS-CoV-2 from January to December 2021 were analyzed. Diagnostic confirmation was obtained through rapid antigen tests in 93.4% of cases, RT-PCR in 6.6%, and a combination of both in 1.6%. Reinfection with SARS-CoV-2 occurred in 0.5% of the study population, with an average of two infections within the year.

The median age was 35 years (range: 1–91). Of the total population, 50.68% were male and 49.32% female. Among women, 4.1% were pregnant, 1.07% were breastfeeding, and 0.19% were in the postpartum period. The median time from symptom onset to clinical consultation was 2 days (range: 0–34).

**Figure 1:**
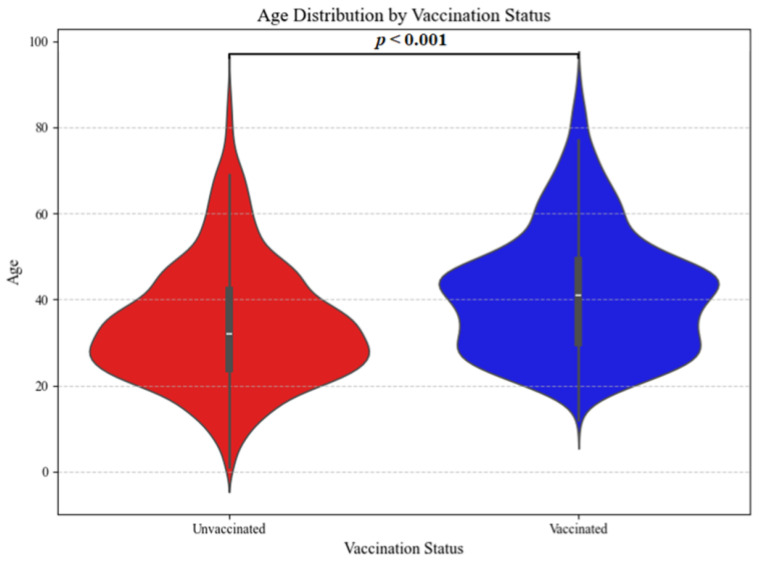
Age distribution among vaccinated and unvaccinated patients.

Age distribution by vaccination status showed significant differences: both the mean and median were higher among vaccinated patients (41.5 vs. 34.2 years, respectively). These differences were statistically significant according to both Welch’s *t*-test and the Mann–Whitney U test (*p* < 0.001) (Figure 1).

Among the study population, 31.45% were vaccinated at the time of diagnosis, while 22.8% of the total had received at least one vaccine dose; 68.54% were unvaccinated at the time of infection. The most frequently administered vaccine was Sinovac (12.4%), followed by AstraZeneca (10.7%), Pfizer (5.2%), Sinopharm (1.2%), CanSino (1.1%), Novavax (0.3%), and Moderna (0.6%).

When evaluating the baseline characteristics of the population, the sex distribution showed a higher proportion of women in the vaccinated group and more men in the unvaccinated group (*p* = 0.003). Hospitalization, mortality, and comorbidity data are summarized in Table 1. Significant differences in comorbidities were observed between groups: hypertension and diabetes mellitus were more common among vaccinated individuals (16.28% and 12.30%, respectively) compared to the unvaccinated group (10.85% and 8.10%; *p* = 0.0001 for both). Obesity was also more prevalent in the vaccinated group (9.62% vs. 7.0%; *p* = 0.002). In contrast, other comorbidities—such as smoking, pneumonia, asthma, immunosuppression, and cardiovascular disease—did not show significant differences between groups (*p* > 0.05). Among the less common conditions, chronic liver disease showed a statistically significant difference despite its low overall prevalence (*p* = 0.035).

**Figure 2:**
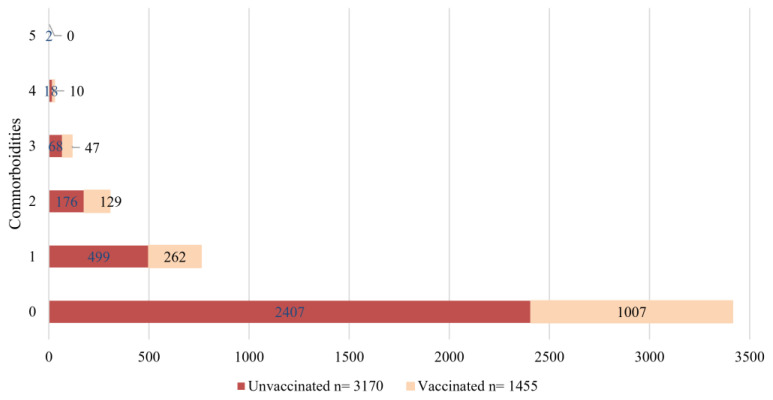
Distribution of the number of comorbidities in vaccinated vs. unvaccinated individuals with confirmed SARS-CoV-2 infection.

When analyzing the distribution of comorbidities by vaccination status, most individuals had no comorbidities, both in the unvaccinated group (75.93%) and in the vaccinated group (69.2%). The remaining individuals in each group had at least one comorbidity. Notably, only two unvaccinated individuals presented with five comorbidities (Figure 2).

The analysis of symptoms between vaccinated and unvaccinated individuals revealed significant differences, particularly in the most common clinical manifestations. Fever, headache, and cough were more frequent among vaccinated individuals compared to the unvaccinated group (*p* < 0.0001 for all comparisons). Less common symptoms, such as dyspnea and chest pain, also showed a significantly higher prevalence in vaccinated individuals (*p* = 0.002 for both). Additionally, other symptoms, including sore throat, general malaise, myalgia, and rhinorrhea, showed statistically significant differences between groups (*p* < 0.0001) (Table 2).

A subgroup analysis was conducted among vaccinated individuals to compare symptom patterns by vaccine type. Recipients of RNA-based vaccines (n = 205) and viral vector-based vaccines (n = 1250) showed no statistically significant differences in symptom frequency. Fever was slightly more frequent in the viral vector group (71.9% vs. 65.8%; *p* = 0.0885), while other symptoms yielded *p*-values > 0.27. No hospitalizations or deaths were observed in either group (*p* > 0.05, Fisher’s exact test). A separate comparison was performed to assess symptom differences according to the number of vaccine doses. Individuals who received one dose (n = 831) had a higher frequency of fever (74.4% vs. 65.7%; *p* = 0.0003), cough (77.0% vs. 69.3%; *p* = 0.0008), and headache (81.6% vs. 76.6%; *p* = 0.0183) compared to those who received two or more doses (n = 624). No statistically significant differences were observed for the remaining symptoms.

The hospitalization rate was 22 per 1000 patients. Among the 4625 individuals analyzed, 153 (3.3%) developed pneumonia, while 104 (2.2%) required secondary-level medical care. Among hospitalized patients, 98.1% (102/104) presented with hypoxemia. Oxygen supplementation was administered via nasal cannula in 28% of cases and by reservoir mask in 55%, while only one patient required CPAP and 16% underwent endotracheal intubation. Hospitalization was required in 76 (2.39%) unvaccinated and 28 (1.9%) vaccinated individuals. No significant association was found between hospitalization and vaccination status (χ^2^ = 1.015; *p* = 0.314).

A bivariate logistic regression analysis was performed to investigate the probability of hospitalization based on the symptoms listed in Table 2 and vaccination status among individuals with confirmed SARS-CoV-2 infection. Dyspnea emerged as a key symptom significantly associated with hospitalization. In unvaccinated patients, a strong association was observed (OR: 8.49; 95% CI: 4.20–17.15), while the risk was also elevated in vaccinated individuals (OR: 7.72; 95% CI: 3.80–15.70).

**Figure 3:**
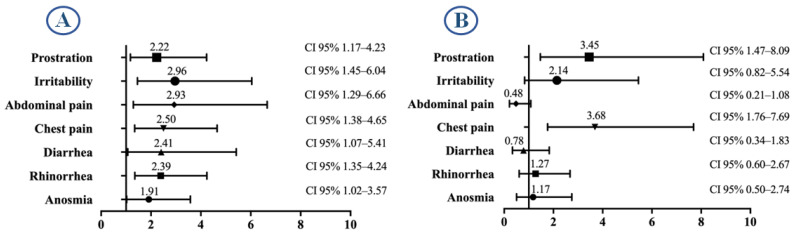
Risk associated with key symptoms related to hospitalization. (**A**) Unvaccinated patients; (**B**) vaccinated patients.

The comparative analysis between vaccinated and unvaccinated patients revealed differences in the association of certain complications with hospitalization. In unvaccinated individuals, symptoms such as prostration, irritability, and abdominal pain, among others, showed a strong association with hospitalization risk. In vaccinated patients, prostration and chest pain were also significantly associated with hospitalization, with higher odds ratios compared to the unvaccinated group. Figure 3 presents the risk associated with the most relevant hospitalization-related symptoms by vaccination status.

To analyze the probability of hospitalization in relation to comorbidities by vaccination status, a stratified logistic regression was performed. Among unvaccinated patients, a higher probability of hospitalization was observed (Nagelkerke R2: 0.856; *p* = 0.0001) in the presence of smoking (OR: 4.75; 95% CI: 2.10–10.72), obesity (OR: 3.85; 95% CI: 1.90–7.78), and hypertension (OR: 2.94; 95% CI: 1.42–6.09). In the vaccinated group (Nagelkerke R2: 0.828; *p* = 0.0001), diabetes mellitus (OR: 3.62; 95% CI: 1.58–8.31) and hypertension (OR: 2.88; 95% CI: 1.51–5.48) were significantly associated with hospitalization risk.

A logistic regression model was fitted with hospitalization as the dependent variable, adjusted for age, sex, and COVID-19 vaccination status. The model showed that older age was associated with higher odds of hospitalization (OR = 1.09, 95% CI: 1.05–1.13), while vaccination was associated with significantly lower odds (OR = 0.38, 95% CI: 0.26–0.55). No significant association was observed for sex. Attempts to incorporate comorbidities such as obesity, diabetes, and hypertension led to multicollinearity and singular matrix issues, likely due to low event frequency in certain subgroups.

**Figure 4:**
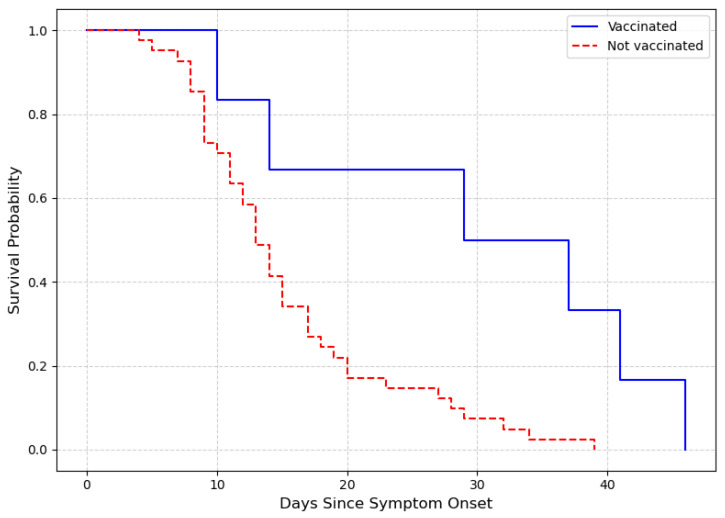
Adjusted survival curves comparing vaccinated and unvaccinated individuals over a 50-day period, based on the Cox proportional hazards model adjusted for age.

A Cox proportional hazards model adjusted for age was conducted to evaluate the association between vaccination status and time to death among confirmed COVID-19 cases. The analysis included 47 deceased individuals (1.0% of the study population) with valid onset and death dates, of whom 34 were unvaccinated and 13 were vaccinated. After adjustment for age, vaccination was associated with a lower risk of death (HR = 0.24; 95% CI: 0.08–0.71; *p* = 0.01) (Figure 4).

## 4. Discussion

Our study shows differences in clinical presentation, comorbidities, and outcomes between vaccinated and unvaccinated patients with laboratory-confirmed COVID-19 in 2021. Contrary to expectations, we observed a higher frequency of symptoms such as fever, headache, cough, sore throat, and myalgia among vaccinated individuals compared to the unvaccinated.

This pattern may reflect a partially effective immune response that reduces severity without preventing the occurrence of mild systemic symptoms. Moreover, the higher prevalence of symptoms in the vaccinated group could be explained by uncontrolled factors in this analysis, such as the significantly older average age and the higher prevalence of comorbidities in this group, partly attributable to the vaccination prioritization policies implemented in 2021 [27,28,29]. It is also possible that a bias occurred whereby vaccinated patients with symptoms sought medical attention more frequently due to concerns about vaccine efficacy or their underlying health status [30,31].

A particularly relevant finding was the higher frequency of anosmia (28.18% vs. 24.04%, *p* = 0.003) in vaccinated patients, contrasting with previous studies that primarily associated this symptom with infections in unvaccinated individuals [32]. Regarding comorbidities, our results identified a higher prevalence of hypertension, diabetes, and obesity in the vaccinated group, consistent with the prioritization of these at-risk groups in vaccination programs [33]. The analysis revealed that these conditions were independent risk factors for hospitalization regardless of vaccination status, although with varying magnitudes of association.

The absolute prevalence of obesity (7.8%) and smoking (5.9%) in our cohort was lower than expected based on population-level estimates. However, these figures are consistent with national data from confirmed COVID-19 cases in Mexico, which report 9.59% for obesity and 5.41% for smoking as of June 25, 2023 [34]. These are standard clinical variables that should be systematically documented in the records. The discrepancy suggests underreporting at the time of case registration, which may have attenuated observed associations.

Both obesity and type 2 diabetes are characterized by chronic low-grade inflammation, which may contribute to immune dysregulation and increased severity of COVID-19. This proinflammatory state has been associated with impaired innate and adaptive immune responses, as well as a higher risk of cytokine dysregulation and complications during infection [35,36]. These mechanisms may help contextualize the elevated hospitalization risk observed in these groups.

A strong association between smoking and hospitalization was observed in unvaccinated individuals (OR: 4.75), whereas the association was weaker among the vaccinated. However, the effects of formal interaction between smoking and vaccination status were not assessed, and thus no effect modification can be established. A study conducted by Paleiron et al. in a confined cohort of unvaccinated young adults reported a lower infection rate and reduced frequency of respiratory symptoms among active smokers [37]. Although their findings did not include severe clinical outcomes or the effect of vaccination, they illustrate the complexity of the relationship between smoking and COVID-19, underscoring the need for studies that examine this interaction in broader and more diverse populations.

To better understand potential confounding effects, we constructed a multivariable model adjusting for age, sex, and vaccination status. The model was statistically valid and consistent with prior findings. However, the inclusion of clinical comorbidities such as diabetes, hypertension, and obesity was not feasible due to collinearity and singular matrix errors, arising from low hospitalization rates in several strata. In this context, it has been noted that structured approaches to confounder selection—grounded in causal reasoning rather than statistical significance—can reduce bias and improve model validity [38,39]. Directed acyclic graphs (DAGs) have been used to identify minimally sufficient adjustment sets and to avoid inappropriate control of colliders or mediators [39]. However, in our study, the use of DAGs was not feasible due to limitations in temporal ordering, sparse data in key strata, and the inability to include all relevant confounders. Despite the limitations in including additional covariates, the adjusted models yielded stable and clinically relevant estimates, reinforcing the protective role of vaccination.

Although chronic liver disease was rare in our population (0.34% in vaccinated vs. 0.12% in unvaccinated, *p* = 0.035), it was the only low-prevalence comorbidity that showed a statistically significant difference between groups. While its low frequency limits its epidemiological weight in this analysis, its identification highlights the importance of considering less common comorbidities in the clinical characterization of COVID-19. This observation becomes more relevant when considering two key aspects: first, the hepatic tropism of SARS-CoV-2 and the mechanisms of liver injury described even in patients without preexisting liver disease [40], and second, the possibility that individuals with chronic liver disease—particularly those with cirrhosis or undergoing immunosuppressive treatment—may develop attenuated immune responses to vaccination due to immune dysfunctions associated with their underlying condition [41]. These findings suggest that even low-prevalence comorbidities may have relevant clinical implications in certain contexts, such as post-vaccination follow-up or risk assessment during future epidemic waves.

The overall hospitalization rate of 2.2% was considerably lower than that reported in early pandemic studies [42,43], likely reflecting improvements in outpatient management and more refined hospitalization criteria by 2021. Although unadjusted comparisons showed no statistically significant differences in hospitalization or mortality between vaccinated and unvaccinated individuals (Table 1), our adjusted analyses revealed that vaccination was a significant protective factor for both outcomes. These findings align with international evidence demonstrating the protective effect of vaccination against severe COVID-19, even in the context of partial coverage and diverse real-world conditions [44,45,46].

The identification of dyspnea as a strong predictor of hospitalization in both groups reinforces its clinical utility as an early marker of severity. The odds ratios were similar (vaccinated: 7.72; unvaccinated: 8.49), with overlapping confidence intervals, indicating no statistically significant difference between groups. This finding supports the use of dyspnea as a key symptom in triage and clinical decision-making. Furthermore, it has been described that this symptom may persist even after clinical recovery, with functional implications requiring multidisciplinary evaluation, as documented in recent studies on post-COVID sequelae [47].

Although both groups presented similar clinical profiles, unvaccinated individuals concentrated a greater number of characteristics significantly associated with hospitalization risk. This finding has direct implications for developing triage algorithms and decision-making regarding outpatient versus inpatient management in the current endemic phase [48].

The analysis of comorbidity distribution showed that between 70% and 75% of patients had no preexisting clinical conditions, regardless of vaccination status. This highlights the need to reconsider risk models that assume comorbidities as the primary determinant of clinical progression, particularly in contexts where other factors may play a more significant role in healthcare demand [49].

Our results indicate that the reinfection rate (0.5%) was relatively low in 2021, which may reflect a combined protective effect of vaccination and prior infection. Although our study did not directly assess the presence or impact of hybrid immunity, it may have contributed to this pattern. Studies such as that by Altarawneh et al. have demonstrated that the protection conferred by hybrid immunity is substantially greater than that of either vaccination or natural infection alone [50].

The 31.45% vaccination coverage observed in our population during 2021 represents a key transitional phase between the pre-immunization stage and generalized vaccine coverage. This unique context allows for the analysis of epidemiological and clinical patterns during an intermediate phase between an unmitigated pandemic and widespread vaccination, which may be useful for future public health emergencies and the implementation of large-scale preventive interventions, as highlighted by the WHO in its pandemic preparedness recommendations [51].

Future studies should incorporate stratified analyses by time since vaccination, vaccine type, complete versus incomplete schedules, and viral variant characterization to refine our understanding of vaccine effectiveness in different clinical and epidemiological contexts. Additionally, the evaluation of specific immunological biomarkers could help identify correlates of protection and explain the observed differences in clinical presentation between vaccinated and unvaccinated individuals [52].

This study has relevant limitations. Its retrospective design precludes the establishment of causal relationships, and the predominant use of rapid antigen tests may have introduced classification bias. Although we were able to adjust for key confounders such as age and vaccination status through a multivariable model, comorbidities could not be included due to instability in parameter estimation, likely caused by low event frequency and multicollinearity. These constraints prevented full adjustment for all potential confounders, and residual confounding cannot be entirely ruled out [39,53]. Moreover, the generalizability of the results is restricted by the single-center design; the lack of stratification by number of vaccine doses, time since vaccination, and vaccine type; and the absence of data on SARS-CoV-2 exposure history, pharmacological prevention measures, circulating viral variants, or patients’ socio-economic status—all of which may have influenced clinical outcomes and the interpretation of effects on hospitalization and mortality.

Despite these limitations, the findings offer valuable insights into the differential clinical behavior of COVID-19 according to vaccination status in real-world care settings. The results support the importance of maintaining targeted symptom surveillance in vaccinated patients, with special attention to dyspnea, prostration, and chest pain as predictors of clinical progression. They also underscore the need for reinforced preventive strategies in patients with specific comorbidities—particularly diabetes and hypertension—even after vaccination.

## 5. Conclusions

This study highlights differences in clinical presentation and comorbidity profiles between vaccinated and unvaccinated patients diagnosed with COVID-19. Although a higher frequency of symptoms was observed in vaccinated individuals, this may be related to uncontrolled factors such as vaccination prioritization and the distinct clinical profiles of the vaccinated group. Dyspnea and prostration were identified as key predictors of hospitalization, underscoring the need for targeted clinical monitoring in vaccinated populations. While unadjusted comparisons showed no significant differences in hospitalization and mortality, adjusted analyses revealed a significant protective effect of vaccination, supporting the role of immunization in reducing severe outcomes. Based on these findings, it remains essential to maintain reinforced preventive strategies in patients with comorbidities such as diabetes and hypertension, as well as for individuals with a history of smoking, regardless of vaccination status.

## Figures and Tables

**Table 1:** Baseline characteristics, comorbidities, hospitalization, and mortality in vaccinated and unvaccinated individuals.

Characteristic	Unvaccinated n (%) n = 3170	Vaccinated n (%) n = 1455	*p* ^†^
Age (min–max)	32 (1–91)	41 (12–91)	0.0001
Men	1653 (52.1%)	691 (47.5%)	
Women	1517 (47.9%)	764 (52.5%)	0.003
Hospitalization	76 (2.4%)	28 (1.9%)	0.314
Mortality	34 (1.1%)	13 (0.9%)	0.573
Hypertension	344 (10.85%)	237 (16.28%)	0.0001
Diabetes mellitus	257 (8.10%)	179 (12.30%)	0.0001
Obesity	222 (7.0%)	140 (9.62%)	0.002
Smoking	111 (3.50%)	50 (3.43%)	0.911
Pneumonia	107 (3.37%)	46 (3.16%)	0.706
Asthma	57 (1.79%)	23 (1.58%)	0.599
Immunosuppression	32 (1.00%)	22 (1.51%)	0.140
Cardiovascular disease	26 (0.82%)	9 (0.61%)	0.462
Renal disease	25 (0.78%)	11 (0.75%)	0.907
Neurological disorder	24 (0.75%)	5 (0.34%)	0.098
HIV ^δ^	8 (0.25%)	8 (0.54%)	0.174
Cancer ^δ^	7 (0.22%)	5 (0.34%)	0.535
Chronic liver disease ^δ^	2 (0.12%)	5 (0.34%)	0.035
Hemolytic anemia ^δ^	4 (0.12%)	1 (0.06%)	1
Tuberculosis ^δ^	3 (0.09%)	0 (0%)	0.556

^†^: χ^2^ test; ^δ^: Fisher’s exact test.

**Table 2:** Frequency of symptoms in vaccinated vs. unvaccinated individuals with confirmed SARS-CoV-2 infection.

Symptom	Unvaccinated n (%) n = 3170	Vaccinated n (%) n = 1455	*p* ^†^
Fever	1881 (59.34%)	1026 (70.52%)	0.000
Cough	1823 (57.51%)	1079 (74.16%)	0.000
Headache	2004 (63.22%)	1150 (79.04%)	0.000
Sore throat	1601 (50.50%)	943 (64.81%)	0.000
General malaise	1358 (42.84%)	730 (50.17%)	0.000
Myalgia	1692 (53.38%)	963 (66.19%)	0.000
Arthralgia	1575 (49.68%)	915 (62.89%)	0.000
Prostration	404 (12.74%)	175 (12.03%)	0.5246
Rhinorrhea	1454 (45.87%)	916 (62.96%)	0.000
Chills	1166 (36.78%)	647 (44.47%)	0.000
Abdominal pain	459 (14.48%)	235 (16.15%)	0.1516
Conjunctivitis	196 (6.18%)	143 (9.83%)	0.000
Dyspnea	279 (8.80%)	171 (11.75%)	0.002
Cyanosis	12 (0.38%)	4 (0.27%)	0.7736
Diarrhea	516 (16.28%)	302 (20.76%)	0.0002
Chest pain	428 (13.50%)	246 (16.91%)	0.0027
Tachypnea	12 (0.38%)	4 (0.27%)	0.7736
Irritability	267 (8.42%)	134 (9.21%)	0.4083
Rhinitis	87 (2.74%)	50 (3.44%)	0.2319
Anosmia	762 (24.04%)	410 (28.18%)	0.003
Dysgeusia	783 (24.70%)	396 (27.22%)	0.074

^†^: χ^2^ test.

## Data Availability

The data presented in this study are not publicly available due to ethical restrictions and institutional regulations regarding the use of patient medical records. Access to the dataset may be considered upon reasonable request and with permission from the Instituto Mexicano del Seguro Social (IMSS).
